# Prevalence of *Vibrio cholerae* in Coastal Alternative Supplies of Drinking Water and Association with *Bacillus*-Like Spore Formers

**DOI:** 10.3389/fpubh.2018.00050

**Published:** 2018-02-26

**Authors:** Md. Asaduzzaman Shishir, Md. Al Mamun, Md. Mahmuduzzaman Mian, Umme Tamanna Ferdous, Noor Jahan Akter, Rajia Sultana Suravi, Suvamoy Datta, Md. Ehsanul Kabir

**Affiliations:** ^1^Centre for Advanced Research in Sciences, University of Dhaka, Dhaka, Bangladesh; ^2^Department of Microbiology, Primeasia University, Dhaka, Bangladesh; ^3^Department of Microbiology, University of Dhaka, Dhaka, Bangladesh; ^4^ISRT, University of Dhaka, Dhaka, Bangladesh; ^5^Emirates Bird Breeding Center for Conservation (EBBCC), Bukhara, Uzbekistan

**Keywords:** *Vibrio cholerae*, Mathbaria, pond sand filter, alternative drinking water, *Bacillus*-like spore formers

## Abstract

The scarcity of hygienic drinking water is a normal phenomenon in the coastal areas of Bangladesh due to the high salinity of ground water. The inhabitants of this locality, therefore, live on alternative supplies of water including rain-fed pond water, and rainwater with persistent complex microbial interactions therein, often contaminated with life-threatening pathogens. Hence, this study was aimed at analyzing the prevalence of *Vibrio cholerae* (*Vc*) in the alternative drinking waters of Mathbaria, a coastal subdistrict neighboring the Bay of Bengal, the efficacy of pond sand filter (PSF) and the co-association among *Bacillus*-like spore formers (Sf) and *Vc*. *Vc* presumably entrapped into the membrane filter was enriched in alkaline peptone water medium and was isolated on selective thiosulfate-citrate-bile salts-sucrose and taurocholate-tellurite-gelatin agar media. They were finally identified by immunochromatographic one step rapid test and serology test. A total of 26% *Vc* positive samples were obtained out of 100 [ponds—48, household (HH)—29, and PSFs—23] where 13% cases were pathogenic (*Vc* O1) and 13% were non-pathogenic (*Vc* non-O1/non-O139). The distribution of *Vc* as observed was 33, 26, and 13.8% in waters derived from pond surface, PSF, and HH reservoirs, respectively, and for pathogenic type, it was 62.5%, 50%, and nil, respectively. Although none of the samples was identified with pathogenic *Vc* O139, the statistics represents a significant and augmentative risk of cholera outbreak in the focused area. The antibiotic sensitivity pattern in this study resembled the trend observed during last few years for *Vc*. The PSF demonstrated its inability to remove *Vc* from any of the samples and in addition, the filter itself was evidenced to be the source of pathogens and spores in further contamination and transmission. The development of biofilm in the PSF could be hypothesized as the reservoir in contaminating pathogen-free water samples. From the test of homogeneity, the risk levels of alternative water sources were estimated equal regarding *Vc*. Simultaneously, it was determined statistically that the prevalence of *Vc*, by no means, is influenced by *Bacillus*-like Sf be it for pond surface, HH, or PSF derived water.

## Introduction

**Figure 1:**
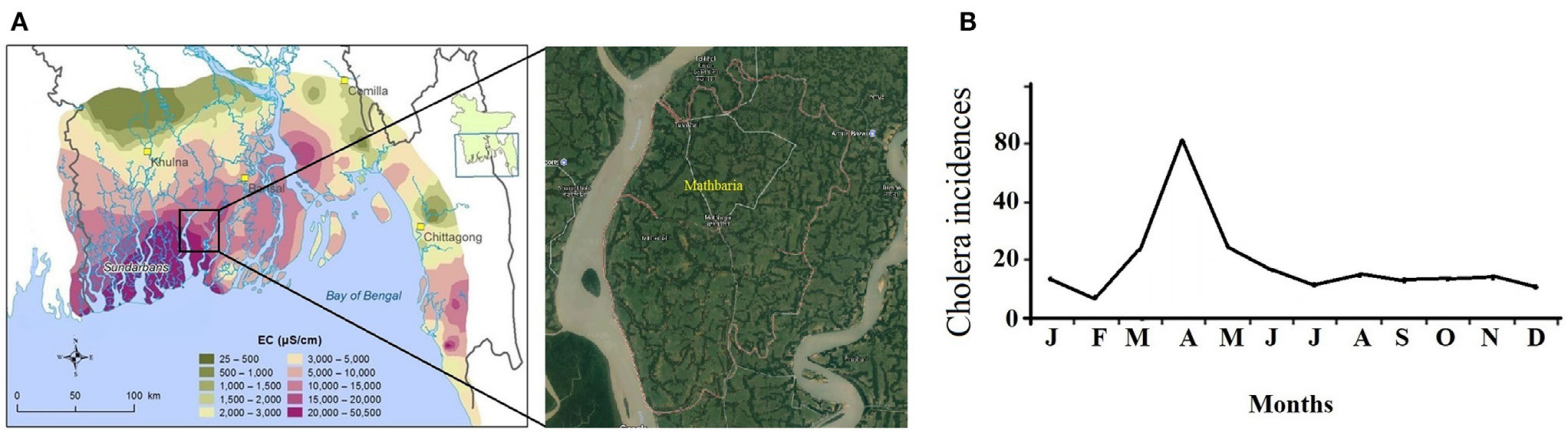
**(A)** Groundwater salinity distribution in coastal Bangladesh [electrical conductivity (EC)] (courtesy: Bangladesh Water Development Board). **(B)** Monthly climatology of cholera incidences in Mathbaria (8).

Due to the complex hydrogeological conditions, rise in sea level, and high salinity in groundwater, 19 coastal districts of Bangladesh were identified as problematic areas, and these regions comprise areas of 47, 211 km^2^ where 28% of the country’s total population (35 million people) resides (1). Certain areas in those districts with their unusable water sources like shallow and deep tube wells usually depend on alternative water supplies. The drinking water—however in these areas—is obtained by direct harvesting of rainwater and from the natural reservoirs, ponds, lakes, etc., where rainwater accumulates in (2, 3). But the pond water in Bangladesh is heavily contaminated with fecal coliforms (FCs), and other pathogenic bacteria (1) and several previous cholera outbreaks also represent the facts (4, 5, 6). Cholera pandemics was first evidenced in the Gangetic delta of the Indian subcontinent and then in other continents (6, 7). In the Bengal delta, the cholera outbreaks are subject to strong spatiotemporal influences, and its occurrences follow the pattern spatially from coast to inland and seasonally from spring to fall. The estuarine river corridors in the coastal area serve as the gateways of planktonic *Vibrio cholerae* during the spring when plankton rich marine water intrudes into the estuary due to the drought mediated reduction in downstream flow of fresh water. The ensuing increase in estuarine salinity creates a natural incubation facility for the bacterium to proliferate and disseminate along the river corridors, which upon human involvement, leads to coastal cholera outbreak (8). In case of extreme droughts, the salinity front might travel up to 100 km into the inland freshwater spreading the pathogen. From there, the *Vc* pathogen is disseminated across the country by inundation due to heavy precipitation during the fall and proliferate in the water logged area after the recession of the flood which is responsible for the fall cholera outbreak (8). Mathbaria, a coastal subdistrict of the district Pirojpur neighboring the Bay of Bengal, known for freshwater scarcity, experience cholera outbreaks predominantly during the spring due to such hydroclimatology and plays an important role in the cholera outbreaks in Bangladesh (Figure 1) (9).

In Mathbaria, the people use the alternative supplies of water and to make the collected water drinkable, pond sand filters (PSFs) are used in purification process whereas the unfiltered water is used for other household (HH) activities. Community based water supply options such as PSFs and community based rain water harvesting systems (CRWHSs) are being promoted for long as these are considered to be efficient for obtaining safe drinking water (1). Several studies from other countries showed that harvested rain water is often contaminated with various pathogenic bacteria and protozoa (10, 11). On the other hand, it was found upon investigations that more than 90% of the samples obtained from PSF in arsenic affected areas of Bangladesh exceeded the microbial specifications set by WHO for thermotolerant coliforms during both dry and wet seasons (3, 12). The FC counts in PSFs were also found to range from 1 to more than 150 colony forming unit (CFU)/100 ml (10).

Toxigenic *Vc* O1 and O139 are causative agents of cholera disease, which cause an acute dehydrating diarrhea (13) and it outbreaks seasonally in the Ganges delta region (9, 14) although the variations in prevalence of the two epidemic serogroups of *Vc*, O1 and O139 are distinct (15). Epidemiological studies on *Vc* O1 and O139, including their emergence, prevalence, and coexistence, have been conducted primarily in Bangladesh and India *via* systematic surveillance (16). The existence of an aquatic environmental reservoir of *Vc* and its association with marine plankton species was firmly established (9, 17, 18) and based on this, satellite remote sensing for chlorophyll, a surrogate for phytoplankton was also proposed to predict cholera outbreaks in the Bengal Delta region (19). In marine environment, the successful colonization of organic matter by *Vc* is dependent on allelochemicals-mediated antagonistic interactions among numerous and diverse bacterial species that reside on the surface of particles. Since, the role of microbial community interacting with each other at an individual scale or a population level, on the abundance of *Vc* is still undefined, a meticulous research in this area might help in revealing the contributing factors involved in this process (20).

Microbial community usually perpetuates through a series of complex interactions among the existing biotic and abiotic factors in different environments. For example, *Vc–*chitin interactions occur at multiple hierarchical levels in the aquatic environment influencing cell metabolic and physiological responses, e.g., chemotaxis, cell multiplication, induction of competence, biofilm formation, commensal and symbiotic relationship with higher organisms, cycling of nutrients, and pathogenicity (21, 22). On the contrary, *Bacillus thuringiensis* was reported to block the quorum sensing in Gram-negative bacteria, especially *Vibrio harveyi* (23) and significant inhibitory activity on biofilm formation was demonstrated against *Vc* by species belonging to *Bacillus cereus* group (could be *B. cereus* or *Bt*), capable of expressing AiiA enzyme (24). Again, *Bt* sp. were reported to exert bacteriocidal activity against *Vc* (25), express highly active chitin degrading enzyme, chitinase (22, 26), synthesize and secrete chitin-binding protein to facilitate microbial attachment to chitin (27) and produce dense biofilms under various conditions (28), etc. These properties of *Bacillus* spp. might influence the existence of *Vc* in the ecosystem in various ways.

It was revealed in a study that the gut microbiota especially *Ruminococcus obeum* limits the colonizing capacity, i.e., the pathogenicity of *Vc* through a novel pathway that does not depend on the AI-2 sensor, LuxP of *Vc* (29). A good number of probiotic products containing diverse *Bacillus* species, are renowned for their efficiency in preventing gastrointestinal disorders and for other beneficial effects like *in vitro* antagonism to the pathogens, competition for nutrients or for adhesion sites, and stimulation of the immune system (30). Understanding the nature of this probiotic effect is complicated since the *Bacillus* species, being allochthonous organism, demonstrate complex microbial interactions within the gastrointestinal tract (31). Thus, it can be perceived that the presence of different *Bacillus* spp. in the aquatic system might also play a pivotal role in the persistence of the water-borne pathogens such as *Vc, E. coli, Shigella*, and *Salmonella* and whether in this process, the quality of water is influenced or not is also very significant.

This study was, therefore, aimed at identifying both *Vc* (pathogenic and non-pathogenic) and spore formers (Sf) including *Bt* from the alternative water supplies to estimate the prevalence of *Vc*, to assess the efficiency of PSF and to evaluate the association of *Bacillus*-like spore formers on the survival of *Vc*, thus the water quality.

## Materials and Methods

### Sample Collection

A total of 100 water samples were collected from different sources [pond surface water (SW)—48, HH water—29, and PSF—23] of Mathbaria subdistrict, Pirojpur. All water samples (500 ml each sample) were collected aseptically in sterile water bottle (leak-proof natural polypropylene wide mouth plastic bottles). The samples were placed in an insulated plastic box within ice after collection and transported to the laboratory.

### Enrichment and Plating

The water samples (40% of 500 ml) were filtered with 0.22 µm polycarbonate membrane filters (Sartorius, USA) and the filter papers were then transferred into sterile alkaline peptone water (APW) (tryptone 10 g/l, sodium cholate 20 g/l; pH 8.6) labeled by sample names. Incubation was then continued at 37°C for 6–8 h for enrichment (32, 33) and approximately 10 µl of the enriched APW broth was streaked onto both thiosulfate-citrate-bile salts-sucrose (TCBS) agar (Eiken, Tokyo, Japan) and taurocholate-tellurite-gelatin agar (TTGA) media (HIMEDIA, India), which were then incubated at 37°C for 18–24 h. The suspected positive samples with specific colony morphology in specific media were then screened for the presence of cytochrome oxidase and gelatinase activity by growing on gelatin agar plate [tryptone 10.0 g/l, trypticase (BBL) 10.0 g/l, gelatin 30.0 g/l, an agar 16.0 g/l; pH: 7.4].

### Immunochromatographic Identification of *Vc* O1 and O139

Immunochromatographic one step rapid test (Dipstick method) was performed for the identification of *Vc* O1 and O139 (34, 35). The qualitative detection of lipopolysaccharide (LPS) antigen of both serotypes was accomplished using monoclonal antibodies specific to *Vc* O1 and O139. In brief, four drops from APW medium after enrichment were taken into a test tube, and a dipstick test strip was placed vertically. After 20 min, the appearance of band in the strip was observed, and the identification of *Vc* O1 and O139 was accomplished in comparison with the positive and negative controls.

### Serogrouping

*Vibrio cholerae* isolates presumed as O1 serotype by Dipstick method was further tested using a serotype-specific monoclonal antibody, Inaba and Ogawa (Remel Europe Ltd., UK) by slide agglutination test (18). In brief, this was performed on a sterile glass slide by resuspending a colony from Gelatin agar plate into sterile physiological saline and subsequently mixing with antibody by tilting the glass slide back and forth. Based on the clumping appearance, the reaction is considered positive.

### Preparation of the McFarland Standard

A 0.5 McFarland standard was prepared by mixing 0.05 ml of 1.175% barium chloride di-hydrate (BaCl_2_·2H_2_O), with 9.95 ml of 1% sulfuric acid (H_2_SO_4_) in a test tube with constant stirring (36). The tube was then sealed tightly and stored in the dark at room temperature.

### Antimicrobial Sensitivity

Susceptibility of the identified *Vc* O1 strains to antimicrobial agents was determined *in vitro* by disc-diffusion method (36) with five different antibiotic disks (azithromycin, ciprofloxacin, erythromycin, tetracycline, and cotrimoxazole). The inocula of the test organisms were prepared by transferring loopful of colony from tryptic soy agar (Sigma, USA) medium into 9 ml of sterile Mueller Hinton broth (Difco, Sparks, MD, USA) and incubated at 37°C for 5 to 6 h. The bacterial cultures were compared with McFarland turbidity standard (10^8^ CFU/ml) and streaked evenly in three directions keeping at a 60°C angle onto the surface of the Mueller Hinton agar plate (10 mm × 40 mm) with sterile cotton swab (36). Surplus suspension was removed from the swab by rotating the swab against the side of the tube before the plate was seeded. After the moisture of the inocula dried up, the antibiotic disks were placed on the agar using sterile forceps and were gently pressed down to ensure contact. For each plate five disks were placed and were placed in a way so that they were no closer than 24 mm. Plates were kept at refrigeration temperature for 30 min for better absorption. During this time microorganisms did not grow but absorption of extracts took place. Negative controls were the disks devoid of any antibiotics. The inoculated plates containing the antibiotic disks were incubated overnight in an upright position at 37°C. The results were expressed as susceptible, intermediate or resistant based on the zone of inhibition in comparison with CLSI standard (12).

### Detection of Sf and *Bt*

Spore formers and *Bt* were detected from the samples following previously described method with some modifications (37). Briefly, about 100 µl of the water samples was aseptically inoculated into 9.9 ml of L-broth (tryptone 10 g/l, yeast extract 5 g/l, and NaCl 5 g/l) supplemented with 0.25 M Na-acetate (pH 6.8) for the detection of *Bt* whereas no supplement was used for the Sf and incubated in an orbital shaker at 30°C and 200 rpm (New Brunswick™ Excella^®^ E25, USA). After 4 h, 0.5 ml of suspension was transferred into a sterile test tube and heat treated for 10 min at 80°C in a water bath. Heat treated suspension was then diluted 10-fold and inoculated onto T_3_-agar medium (1.0 L: tryptone 3.0 g, tryptose 2.0 g, yeast extract 1.5 g, MnCl_2_ 0.005 g, phosphate buffer 50 mM, and agar 15.0 g; pH: 6.8) (38) by spread plate method and incubated at 30°C. In case of appearance of any colony after overnight incubation, incubation period was extended up to 72 h to allow sporulation.

### Statistical Analysis

Statistical analyses were conducted using software R version 3.4.1. The data analysis was performed by appropriate statistical tests where feasible. The Risk analysis of the alternative water sources was carried out by the test of homogeneity of proportions. Association among *Vc* and Sf as well as *Bt* was evaluated by Boschloo’s exact test with binomial model or multinomial model, Pearson’s chi-squared test, Fisher’s exact test, etc., where applicable. Boschloo’s exact test is an unconditional exact test for which the current version of the R package “Exact” (version 1.7) was used.

## Results and Discussions

### Prevalence and Distribution of *Vc* and Sf in Mathbaria

**Figure 2:**
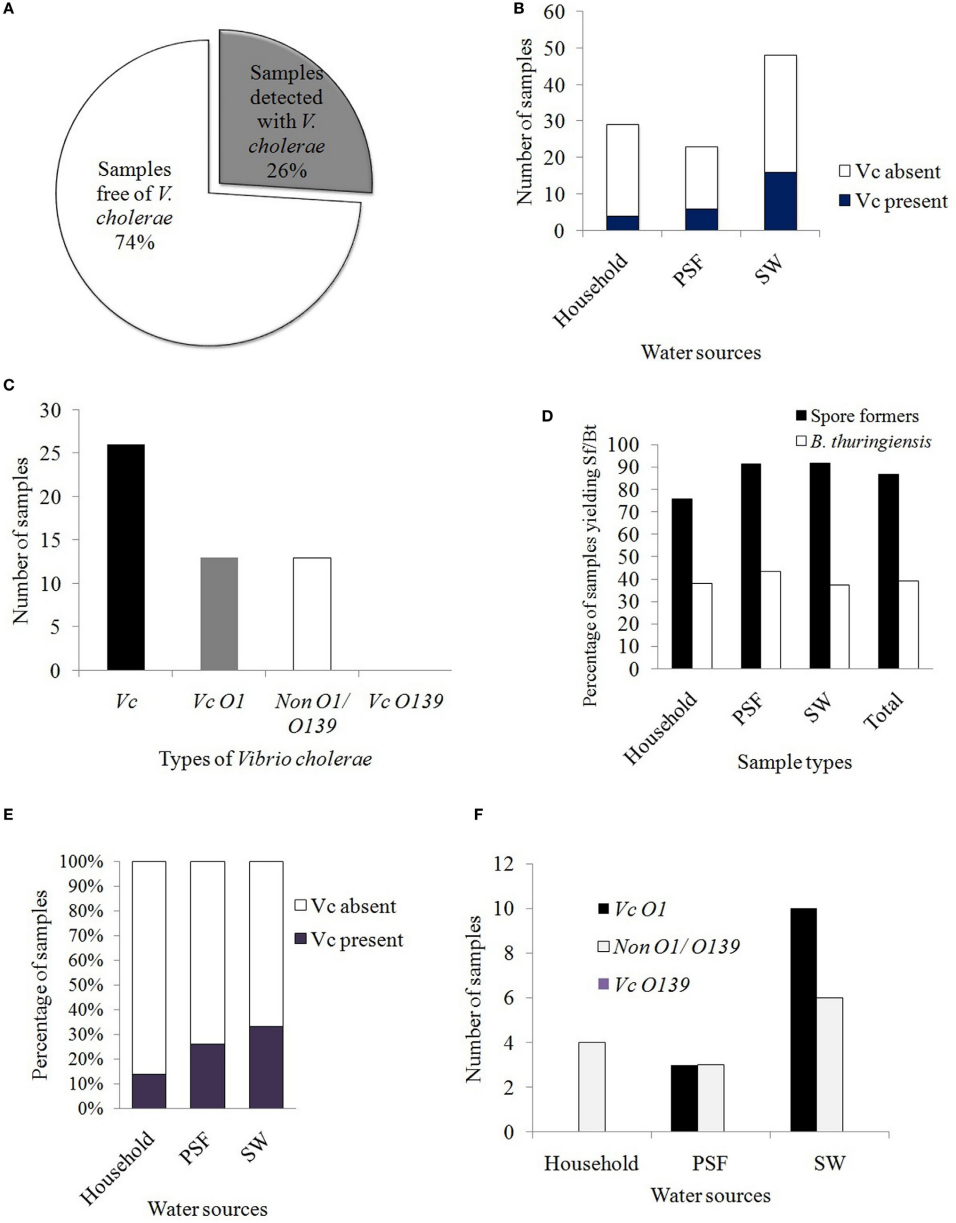
**(A)** Prevalence of *Vibrio cholerae* in the different water samples tested. **(B)** Distribution of *Vc* in the alternative drinking water sources. **(C)** Prevalence of pathogenic and non-pathogenic *Vc* in the tested samples. Bar diagram represents the serotypes of *Vc*. **(D)** Prevalence of spore formers and *Bacillus thuringiensis* in different sources of water in Mathbaria. **(E)** Comparison of water quality on the basis of *Vc* detection rate. **(F)** Distribution of different serotypes of *Vc* in different sources of alternative drinking water.

Following conventional method (colony characteristics on selective media) and rapid dipstick test, it was observed that 26% of the samples (i.e., 26 out of 100 samples) contained *Vc* isolates (Figure 2A). The distribution of *Vc* among different water supplies was estimated to be 13.8, 26, and 33% in waters derived from HH reservoirs, PSF, and pond surface, respectively (Figure 2B). Among the *Vc* identified, 13% were pathogenic (*Vc* O1) and 13% were non-pathogenic (non-O1/O139) (Figure 2C). None of the sample was identified with *Vc* O139 and this was further confirmed by serological test performed with specific antisera.

*Vibrio cholerae* positive samples were identified by TCBS and TTGA selective media following enrichment in APW broth and rapid dipstick test was performed with the *Vc* enriched APW broth to detect the presence of serotypes O1 and/or O139. Among all the samples plated on TCBS and TTGA media after enrichment in APW broth, characteristic growth of *Vc* was observed in both media with 22 samples whereas in 7 cases, growth of *Vc* was observed either in TTGA (3) or in TCBS (4). By the dipstick test and slide agglutination test with specific antisera, four out of seven were further confirmed as *Vc*. In developing countries, crystal VC dipstick has been the reliable test for detection of *Vc* these days and other rapid tests are also available for the LPS signal to be amplified in the APW broth before testing (39). The crystal VC dipstick was reported to possess much higher specificity than previous records (91–98%) and hence, this method was suggested as a promising screening tool for cholera outbreak surveillance in resource-limited settings (34).

Out of 100 water samples collected, 77 were unique since the samples collected from the PSF (23), in fact, represent the pond SWs undergone filtration process in PSF. Among the pathogenic *Vc* O1, 20.8% cases (10 out of 48 samples) were from pond SW, being the maximum, followed by 13% (3 out of 23 samples) from PSFs whereas no pathogenic *Vc* (out of 29 samples) was detected from HH water.

On the other hand, 87% of the water samples yielded Sf and *Bt* was isolated from 39% of the total samples. The distributions among the sources for Sf were 76, 91, and 92% and for *Bt*, were 38, 43, and 37.5% in HH water, PSF water, and pond SW, respectively (Figure 2D).

Cholera outbreaks occur seasonally in the coastal areas of Bangladesh such as Mathbaria, where it is predominant during the spring (from March through May), i.e., the dry season and the inhabitants are most susceptible since the scarcity of drinking water due to the high salinity in the shallow and deep tube-well water, deteriorate the situation (9, 40). The prevalence of *Vc* in season before the spring should be different and the study was, therefore, conducted during the beginning of winter, in the months of November–December to assess the prevalence of *Vc* which in other sense, represents the safety of the alternative drinking waters.

The significant finding here is the detection of pathogenic *Vc* O1 in 13.0% of the total samples in the month of November–December which is half of the total *Vc* positive samples (26). Between March and December 2004, in a study conducted by Alam and others, 99 water samples from Mathbaria yielded 2% *Vc* O1, 1% O139 Bengal, and 41.8% non-O1/O139 isolates where the maximum *Vc* O1 was isolated in the month of December (18), similar to our case. Again, pathogenic *Vc* O1/O139 was not detected from the coastal areas, especially subdistricts, Dacope and Mongla of districts, Khulna and Bagerhat, respectively, as reported in another study (1). In our study, although the overall prevalence seemed to be lower than others, a sign of more protected rain-fed ponds and better hygiene practice among the inhabitants, the detection of more than 20% *Vc* O1 (10 out of 48) in the rain-fed pond SW alone is very alarming. Since, the isolation was accomplished following enrichment in APW broth, and the direct isolation of *Vc* O1 even in cholera endemic areas was reported rare (18, 41), the isolates could be considered as viable but non-culturable. The variation in prevalence especially the increase of *Vc* O1 by a decade indicates the augmentative risk of cholera outbreaks persisting in this region. Whether they are toxigenic or not, should be determined by M-PCR (18) and further investigation for the virulence genes such as, *ctxA* and *rfb*, is required to estimate the risk level.

The ponds, in this study, were selected in a random manner for sample collection, and those were scattered inside and in the surroundings of Mathbaria municipal arena. The ponds in the surroundings were mostly connected to the canals and were also used in utensils washing as well as in bathing purposes. Hence, the tidal waters from the estuarine that intruded into the ponds *via* the canals, might have contributed to the transportation of *Vc* reservoir, i.e., the planktons (17). Again, as the study period was at the outset of winter, gradual reduction in rainfall and shift in temperature could have affected this mangrove forest neighborhood by contributing available nutrients to promote the growth of *Vc* O1. Thus, the difference in locations, variation in temperatures and artifactual contaminations might be the facts behind the variable prevalence of *Vc*. On the other hand, HH water, even though the prevalence of *Vc* was not very high, could be contaminated during rainwater harvesting or from manual collection system, dirty or blocked gutter and lack of initial washing or dirty storage tanks, etc. As the PSF water samples represent the rain-fed pond SW, the variation in prevalence is correlated to the efficiency of the PSF.

Prevalence of non-pathogenic *Vc* in the beginning of winter season was observed to be 12.5% (6 out of 48) and 13% (3 out of 23) in pond SW and PSF water, respectively (Figure 2F). Islam and coworkers reported that non-pathogenic *Vc* (non-O1/non-O139) from different water sources (ponds water, PSFs water, and harvested rain water) during dry and wet seasons were 47 and 100%, respectively, for PSFs whereas for pond SW, those were 95% in both seasons (1). This indicates that the rain-fed ponds of Mathbaria are better protected than those of Dacope and Mongla, and the inhabitants used them more hygienically. Again, since *Vc* non-O1/non-O139 was also reported to be clinically significant since *ctxA, ace*, or *zot* genes were present in few of the tested isolates (41), the PCR detection of virulence genes should be performed with all of the *Vc* positive samples for a better risk analysis.

### Risk Analysis of the Alternative Water Supplies

The prevalence of *Vc* in the water samples from HH, PSF, and rain-fed pond surface was estimated and was compared. It was found that maximum 33% of the water samples from pond surface were identified with *Vc* followed by 26% in PSF and 13.8% in HH water. All *Vc* serotypes (4 out of 4) obtained from HH water were non-pathogenic, non-O1/non-O139 whereas 50% from PSF (3 *Vc* O1 out of 6) and maximum 62.5% (10 *Vc* O1 out of 16) from rain-fed pond SW yielded pathogenic *Vc* O1 (Figure 2E). The risk level of alternative water supplies as revealed in this study indicates that the pond SW was riskier with higher prevalence of both pathogenic and non-pathogenic *Vc* than that obtained from PSF and HH reservoirs.

The estimates of the proportions of *Vc* were 0.33, 0.14, and 0.26 in pond SW, HH water, and PSF, respectively. The test of homogeneity of proportions was conducted, and the null hypothesis was assumed that the proportions of *Vc* estimated from all sources of water tested, were the same, i.e., H_0_: the proportion of *Vc* is the same in each sources, p_1_ = p_2_ = p_3_. And the alternative hypothesis was, H_1_: at least two proportions are unequal. Based on the samples, the rejection of the null hypothesis $\left(\chi_{2}^{2}=\text{3.5877, }p\text{-value}=0.1663\right)$ was failed at 5% level of significance, i.e., the data provide sufficient evidence to conclude that there was no significant difference in the rates of *Vc* from one source to another source. Hence, it could be perceived that

(i) The chances of cholera incidences are almost similar if any of the sources is used in drinking purpose.
(ii) The efficiency of the PSF in reducing the risk of *Vc* is insignificant and compromised in many cases.
(iii) The HH reservoir could be contaminated during washing with contaminated waters, or unwashed reservoirs were used.

*Vibrio cholerae*, both pathogenic and non-pathogenic together, were considered for the test of homogeneity of proportions since the sample size of pathogenic *Vc* was not significant in all three cases, i.e., for HH water, it was nil. Contrarily, the loss of virulence genes in *Vc* O1 (17) and detection in *Vc* non-O1/non-O139 in certain cases (41) emphasized both types to be considered for the statistical analysis. Nevertheless, detection of virulence genes in the metagenomic pool could be useful in providing more insights to compare the alternative water supplies more appropriately.

Regarding the efficiency, it was observed that upon filtration through the PSF, *Vc* was not removed in any cases (*n* = 16) rather persisted in all cases and in 8.33% cases (*n* = 12), the contamination of *Vc* was introduced from the filter itself. This indicates that the filter itself can be the source of pathogens for further contamination and transmission which eventually cannot be considered as safe. The reports of inefficacy, published previously (3, 10, 12), are in agreement with the results obtained. The development of biofilm in the PSF, as also detected earlier in water and stool which served as the reservoir for long-term viability of *Vc* O1 (17), could be similar here for contaminating fresh waters. Chitin particles entrapped in the sand bed could be colonized by *Vc* and utilized with the self-secreted chitinase enzyme for nutrients (21), and biofilm formation thereby might have facilitated their existence. More studies in this connection are required to resolve the factors functioning in such case.

In case of Sf, the PSF was not able to clean up spores from any samples (*n* = 16), i.e., in 100% samples, filtration could not make any difference rather one of the samples was found to be contaminated in this process. Although upon filtration, *Bt*, another spore forming bacilli, was removed from 28.5% of the samples, 33.3% of the samples free of *Bt*, were contaminated with that very organism. The alteration of water quality thereby is also dangerous as among the Sf, there could be pathogens such as *B. cereus* and *Clostridium perfringens* responsible for gastroenteritis and food borne intoxication.

*Bacillus*-like Sf are usually soil dwelling bacteria and since they are ubiquitous, it is very likely that they can be obtained from water too. The presence of *Bacillus thuringiensis* was reported in many habitats of Bangladesh including southern region (basically coastal area), river basin and sandy beach with *Bt* indices (0.87, 0.82, and 0.73, respectively) (37). Sand is an important component of the PSFs, currently in use in Bangladesh, be it coarse or fine and needs scheduled regeneration by washing, chlorination, drying, etc. (42). Change in trophic level due to accumulation of water-borne biotic and abiotic as well as soluble and insoluble components in the sand bed, might facilitate the propagation of *Bacillus*-like Sf and discharge during filtration, a possible cause for contamination of filtered waters with spores.

On the other hand, the practice of regeneration of the PSF sand bed with the appearance of discernible change only, might be responsible for the unpredictable efficiency of PSF. Usage time relevant data and close monitoring for cholera pathogen would be useful in this connection to estimate the shelf life of PSF too. On the other hand, research for the development of simple and rapid regeneration method of sand bed to disrupt the presumptive biofilms would be more feasible, viable, and sustainable.

### Antibiotic Sensitivity Pattern of *Vc*

**Figure 3:**
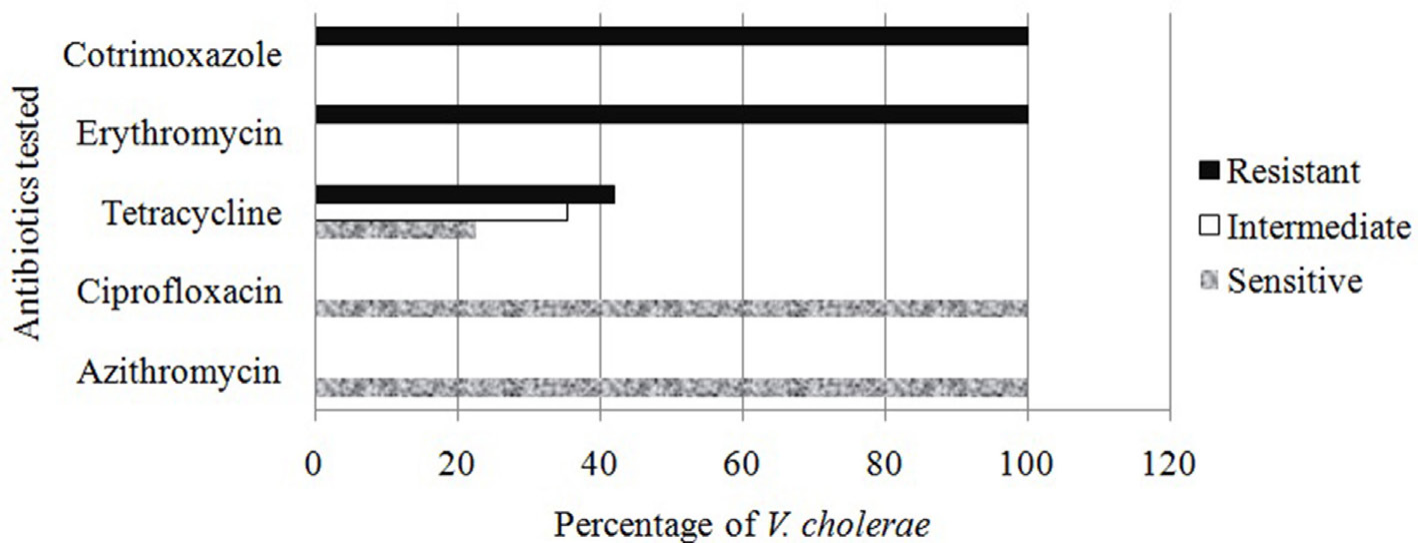
Antibiotic sensitivity study of the *Vibrio cholerae* detected from different water sources.

In antibacterial sensitivity test, it was found that all isolated *Vc* O1 were sensitive (100%) to azithromycin and ciprofloxacin whereas all were resistant to erythromycin and cotrimoxazole. In case of tetracycline, about 42% were resistant, 22.5% were sensitive, and the rest were variable (Figure 3). Antibiotic sensitivity pattern of the *Vc* was observed to be similar to the prevailing trend revealed in last few years of study period (40, 43).

In a recent study, it was observed that susceptibility of *Vc* toward azithromycin and ciprofloxacin was reduced, and 95% sensitivity to CIP was evidenced (40). Extensive and unregulated administration of antibiotic in health issues, veterinary medicines even in animal feeds in Bangladesh might be the source of such resistance. The complete resistant to erythromycin was developed because of its frequent use as a growth promoter in food animal production (44).

### Assessment for Association of *Bacillus*-Like Sf with *Vc*

**Figure 4:**
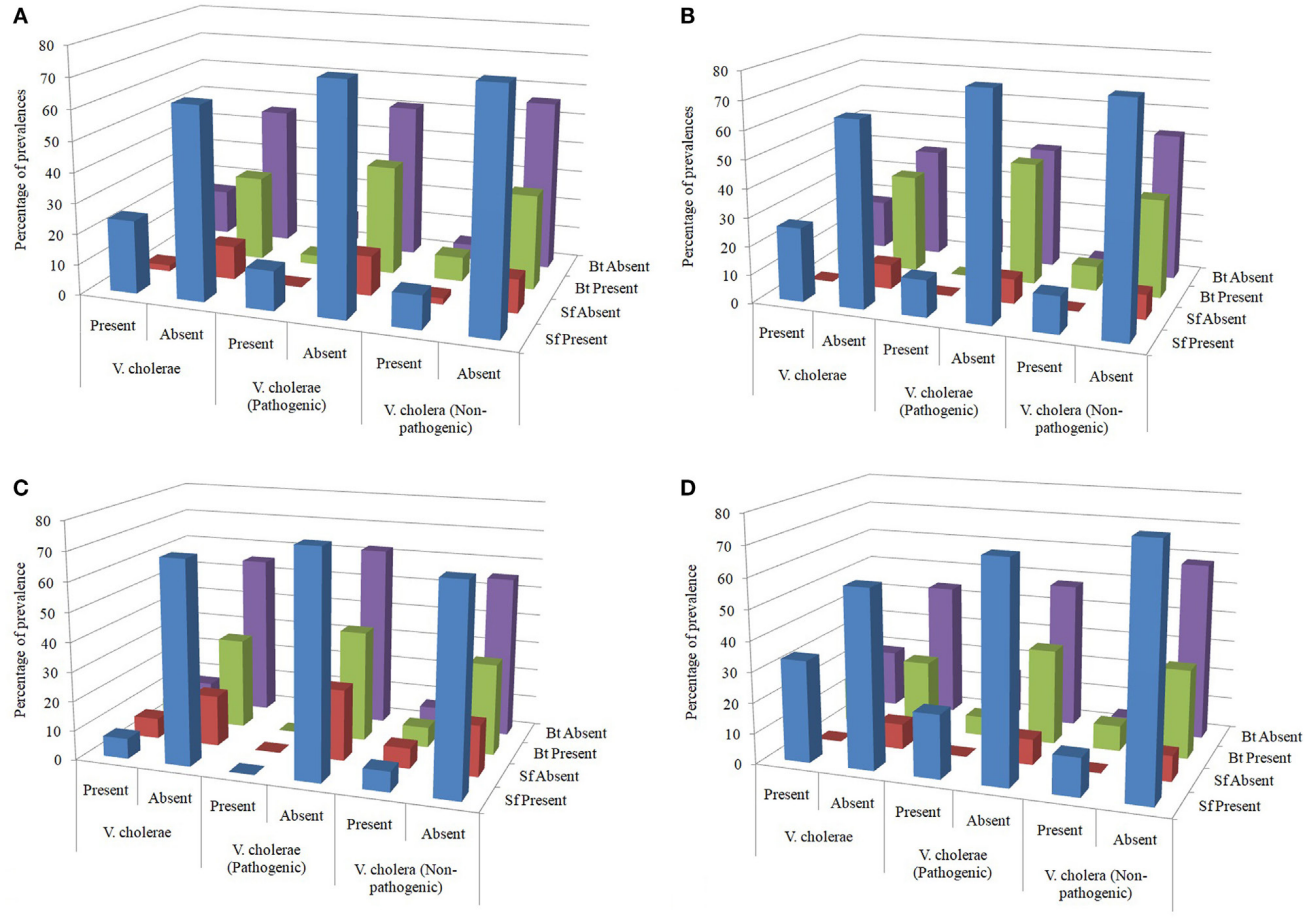
**(A)** Overall distribution of spore formers (Sf) and *Bacillus thuringiensis* in the water samples tested. **(B)** Distribution of Sf and *Bt* in the water samples from pond sand filter. **(C)** Distribution of Sf and *Bt* in the household water samples. **(D)** Distribution of Sf and *Bt* in the water samples of pond surface.

The influence of Sf on the survival of *Vc* was evaluated statistically based on the reciprocal prevalence as several reports of their antagonism against *Vc* are also available. The percentages of presence or absence of Sf and *Bt* in the samples detected with as well as free of *Vc* provided the impression that Sf and *Bt* might have an ecological influence over *Vc* (Figure 4A). The presumption that the survival of *Vc* (both pathogenic and non-pathogenic) might be influenced by the Sf as well as *Bt* was therefore evaluated. Hence, the presence and absence of both Sf and *Vc* as well as *B. thuringiensis* (*Bt*) and *Vc* were tabulated and analyzed statistically to find any association among them. The co-association tests were, therefore, performed separately and together with all water sample types.

Overall prevalence of Sf in samples detected with *Vc, Vc* O1, and *Vc* non-O1/non-O139 were 24, 13, and 11, respectively, out of 100 samples and it was 11, 3, and 8 for *Bt*. Again, in samples free of *Vc, Vc* O1, and *Vc* non-O1/non-O139, prevalence of Sf were 63, 74, and 76 whereas it was 28, 36, and 31 for *Bt*, respectively (Figure 4A).

Table 1 shows the overall and source based results of appropriate association tests between *Vc* (pathogenic and non-pathogenic) and Sf as well as *Bt* (45, 46). Overall test results indicated that no association between Sf and *Vc* (both pathogenic and non-pathogenic) could be found considering all the samples, and the similar outcome was observed in case of *Bt* over *Vc* and its types.

**Table 1:** Results of association test between *Vc* (pathogenic and non-pathogenic) and Sf as well as *Bt*.

Source	Association	Test name	Test statistic	*p*-Value
Overall	Sf vs *Vc*	Boschloo’s exact test (multinomial)	0.5049	0.4566
	Sf vs *Vc* (pathogenic)	Boschloo’s exact test (binomial)	0.2076	0.1609
	Sf vs *Vc* (non-pathogenic)	Boschloo’s exact test (binomial)	0.6757	0.5827
	*Bt* vs *Vc*	Pearson’s chi-squared test	0.1616	0.6877
	*Bt* vs *Vc* (pathogenic)	Boschloo’s exact test (binomial)	0.2407	0.2193
	*Bt* vs *Vc* (non-pathogenic)	Boschloo’s exact test (binomial)	0.1248	0.1010

Pond sand filter	Sf vs *Vc*	Boschloo’s exact test (multinomial)	1	1
	Sf vs *Vc* (pathogenic)	Boschloo’s exact test (binomial)	1	1
	Sf vs *Vc* (non-pathogenic)	Boschloo’s exact test (binomial)	1	1
	*Bt* vs *Vc*	Boschloo’s exact test (multinomial)	0.6600	0.5724
	*Bt* vs *Vc* (pathogenic)	Boschloo’s exact test (binomial)	0.2292	0.2091
	*Bt* vs *Vc* (non-pathogenic)	Boschloo’s exact test (binomial)	0.5596	0.4175

Household water	Sf vs *Vc*	Boschloo’s exact test (multinomial)	0.2381	0.1760
	Sf vs *Vc* (pathogenic)	Fisher’s exact test	0	1
	Sf vs *Vc* (non-pathogenic)	Boschloo’s exact test (binomial)	0.2381	0.1964
	*Bt* vs *Vc*	Boschloo’s exact test (multinomial)	0.6221	0.4903
	*Bt* vs *Vc* (pathogenic)	Fisher’s exact test	0	1
	*Bt* vs *Vc* (non-pathogenic)	Boschloo’s exact test (binomial)	0.6221	0.5169

Pond surface water	Sf vs *Vc*	Boschloo’s exact test (multinomial)	0.2863	0.2334
	Sf vs *Vc* (pathogenic)	Boschloo’s exact test (binomial)	0.5664	0.4491
	Sf vs *Vc* (non-pathogenic)	Boschloo’s exact test (binomial)	1	1
	*Bt* vs *Vc*	Pearson’s chi-squared test	0.4000	0.5271
	*Bt* vs *Vc* (pathogenic)	Boschloo’s exact test (binomial)	0.7222	0.6527
	*Bt* vs *Vc* (non-pathogenic)	Boschloo’s exact test (binomial)	0.1793	0.1369

*Sf, spore formers; Vc, V. cholerae; Bt, B. thuringiensis*.

Whether the association is varied over the sample types or not was also assessed, and the statistical analyses were performed separately for all sample types.

In PSF, prevalence of Sf in samples detected with *Vc, Vc* O1, and *Vc* non-O1/non-O139 were 6, 3, and 3, respectively, out of 23 samples and it was 2, 0, and 2 for *Bt*. Again, in samples free of *Vc, Vc* O1, and *Vc* non-O1/non-O139, prevalence of Sf was 15, 18, and 18 whereas it was 9, 10, and 12 for *Bt* (Figure 4B). Prevalence was expressed in percent in the diagram in all similar cases if not otherwise stated. Table 1 shows the results of association test between various attributes obtained from the PSF water samples. Based on the data, it can be concluded that neither Sf nor *Bt* influence the distribution of *Vc* (both pathogenic and non-pathogenic) in the PSF water.

In HH reservoir, prevalence of Sf in samples detected with *Vc, Vc* O1, and *Vc* non-O1/non-O139 was 2, 0, and 2, respectively, out of 29 samples, and it was 2, 0, and 2 for *Bt*. Again, in samples free of *Vc, Vc* O1, and *Vc* non-O1/non-O139, prevalence of Sf were 20, 22, and 20 whereas it was 9, 11, and 9 for *Bt* (Figure 4C). Table 1 shows the results of association test between various attributes obtained from the HH water sample. The data provide sufficient evidence to conclude that neither Sf nor *Bt* influence the distribution of *Vc* (both pathogenic and non-pathogenic) in the HH water.

In pond SW, prevalence of Sf in samples detected with *Vc, Vc* O1, and *Vc* non-O1/non-O139 were 16, 10, and 6, respectively, out of 48 samples and it was 7, 3, and 4 for *Bt*. Again, in samples free of *Vc, Vc* O1, and *Vc* non-O1/non-O139, prevalence of Sf were 28, 34, and 38 whereas it was 11, 15, and 14 for *Bt* (Figure 4D). Table 1 shows the results of association test between various attributes obtained from the SW sample. Based on the data, it can be concluded that Sf or *Bt* do not influence the distribution of *Vc* (both pathogenic and non-pathogenic) in the pond SW.

In a 2 × 2 contingency table, if no expected frequency is less than 5, a Pearson’s chi-squared test was conducted and on the contrary, an exact test was done (47). In case of both margins being fixed of a 2 × 2 contingency table, Fisher’s exact test was conducted. If single margin was fixed, Boschloo’s exact test was used with a binomial model, and if no margin was fixed, Boschloo’s exact test with a multinomial model was performed (45). Fisher’s exact test was also performed in cases where all cells of a row or column are 0 (48).

The test results of appropriate association, although the impression of correlation could be perceived from graphical representation (Figure 4), revealed that neither the *Bacillus*-like Sf nor the *Bt* has any influence on the prevalence of *Vc*, be in the pond surface, HH reservoir or PSF derived water. The *p*-values in all cases were greater than 0.05 which nullified the chances of any co-association (Table 1).

Usually, *Vc* O1 and O139 remain preponderantly as viable but non-culturable cells in water and culturable cells in biofilm consortia either free-swimming or attached to plankton or other aggregates (17, 18). In this study, only water samples were considered, and no plankton samples were used in isolation of either *Vc* or Sf or *Bt*. Hence, the assessment of association was directed solely to the drinking water of alternative supplies. Although the binary data of presence or absence, analyzed statistically provided no clue of association between the bacteria of interests, estimation of CFU counts could provide a deep insight into any correlation prevailing among them or not. Contrarily, biofilm consortia could be the potential arena where *Vc* could be affected by *Bacillus* spp. due to complex microbial interaction as reported by Augustine and coworkers from their *in vitro* analysis (24). Although it was observed that the introduction or retention of *Vc* from the PSF was occurred in cases where *Bt* was neither present nor introduced and introduction or retention of *Bt* was occurred where *Vc* was absent, a larger sample size associated with the detection of *aiiA* gene in the metagenomes is essential to conclude any *in situ* inhibitory effect against biofilm formation. At the same time, prevalence of *Vc* and other *Bacillus* spp. from plankton and other biofilm harboring aggregates should be determined in this connection.

## Conclusion

Although the fatality rate during a cholera outbreak has been reduced by simultaneous administration of oral saline, intravenous rehydration therapy as well as antibiotic treatment, *Vc* still continues to be a serious threat to the public health in countries like Bangladesh. A routine surveillance system, therefore, for the determination of the prevalence and antibiotic sensitivity of *Vc* as well as the detection of antibiotic resistant genes in the metagenomic pools, could be prognosticative before any eruption to increase awareness among the people and government for a better risk management. In this study, it was revealed that the alternative drinking water supplies of coastal region of Bangladesh, especially Mathbaria, contain both pathogenic and non-pathogenic *Vc* at alarming level. Hence, the ponds should be protected from external contamination with high banks, avoiding bathing, washing of utensils, and disrupting any connection with the canals carrying tidal brackish water from the estuarine. Statistically, no significant differences among the alternative water supplies could be concluded. Rather, the PSFs were found to be completely inefficient in removing *Vc* and *Bacillus* spores and often unpredictable in providing microbiologically safe drinking water. A simple and rapid method to regenerate the sand bed in the conventional PSF and well researched determination of shelf life might help to prevent it from being a source of contamination. Public awareness should be increased in this connection providing sufficient knowledge and training on operation and maintenance of PSF for hygienic water. Although no association was established between *Vc* and *Bacillus* spp. from the alternative drinking water supplies, their prevalence from plankton and other biofilm harboring aggregates should be explored to determine any potential *in situ* interaction. To the best of our knowledge, this is first study conducted to analyze any association between *Vc* and *Bacillus*-like Sf in the alternative supplies of drinking water.

## Author Contributions

MAS designed the research plans, worked in the laboratory too to generate data, analyzed the data, and prepared the manuscript and finalized it. MAM collected the water samples and isolated and identified *V. cholerae*. He also performed serogrouping and prepared certain sections of the manuscript. NA performed all the statistical analysis. MAM, MMM, UF, and RS performed the isolation of the Sf and *Bacillus thuringiensis*. SD co-supervised the research and also provided scrupulous thoughts on the manuscript. MK helped in preparing the manuscript with certain literature review.

## Conflict of Interest Statement

The authors declare that the research was conducted in the absence of any commercial or financial relationships that could be construed as a potential conflict of interest.
